# É Possível Estudar de Forma não Invasiva as Adaptações Hemodinâmicas da Cardiomiopatia Chagásica pela Curva Volume-Tempo Utilizando a Ecocardiografia 3D?

**DOI:** 10.36660/abc.20220284

**Published:** 2022-06-06

**Authors:** José Luiz Barros Pena

**Affiliations:** 1 Faculdade Ciências Médicas de Minas Gerais Belo Horizonte MG Brasil Faculdade Ciências Médicas de Minas Gerais, Belo Horizonte, MG – Brasil; 2 Hospital Felício Rocho Belo Horizonte MG Brasil Hospital Felício Rocho, Belo Horizonte, MG – Brasil

**Keywords:** Ecocardiografia, Tridimensional/métodos, Insuficiência Cardíaca, Cardiomiopatia Chagásica, Volume Diastólico, Pressão Sanguínea

A ecocardiografia tridimensional (3DE) representa uma grande inovação no ultrassom cardiovascular.^1^ O aumento do poder de processamento computacional e os avanços no desenvolvimento de transdutores permitiram a aquisição de estruturas cardíacas de qualquer ponto de vista espacial, sem suposições sobre sua forma. Estudos recentes demonstraram que, quando os tamanhos das câmaras cardíacas são quantificados pelo 3DE, seus volumes são semelhantes aos obtidos pela ressonância magnética cardíaca em comparação com a ecocardiografia bidimensional (2DE).^2,3^ A utilidade da 3DE foi particularmente demonstrada principalmente em imagens anatômicas realísticas de valvas cardíacas e na orientação e monitoramento de procedimentos cardíacos.^4^

A 3DE permite o cálculo do volume do ventrículo esquerdo (VE) ao longo do ciclo cardíaco, possibilitando a construção de uma curva volume-tempo. Esse método é mais preciso que a 2DE, pois a construção do volume ventricular esquerdo é realizada por meio da análise de centenas de pontos na borda do endocárdio. Nenhum plano específico ou modelo geométrico é necessário para descrever a estrutura complexa do VE. Neste artigo, Pinto et al.,^5^ testaram a hipótese de estudar as adaptações hemodinâmicas da cardiomiopatia chagásica não invasiva utilizando a curva volume-tempo gerada pelo 3DE.^5^ Eles geraram um polinômio ajustado à curva de volume do VE por meio de software específico. O objetivo foi apresentar um estudo transversal avaliando a função do VE, comparando curvas de volume em 20 pacientes com cardiomiopatia chagásica (CC) e 15 controles saudáveis pareados por sexo e idade.

Os pacientes CC apresentaram maiores volumes diastólico e sistólico final do VE e fração de ejeção do VE menor do que o grupo controle. No entanto, o volume sistólico e o fluxo máximo de ejeção durante a sístole, QS, foram semelhantes entre os grupos. Uma forte correlação entre fluxo e volumes sistólicos foi demonstrada, Rs=0,91, p<0,001.

O grupo CC apresentou uma relação volume diastólico final QS/VE inferior ao controle. A relação volume diastólico final QS/VE apresentou forte correlação com a fração de ejeção, Rs=0,89, p<0,001.

O fluxo máximo nas fases de enchimento inicial e passivo, QE e durante contração atrial, QA, foi semelhante entre pacientes e controles.

Embora os pacientes CC apresentassem disfunção sistólica grave do VE com fração de ejeção de 30%, os volumes sistólicos foram semelhantes aos controles.^5^

Qualquer VE com uma fração de ejeção baixa, mas com volume diastólico final aumentado ejeta a mesma quantidade de sangue que um VE com volume diastólico final e fração de ejeção normais. Essa diferença ocorre devido à preservação do mecanismo de Frank-Starling em pacientes com CC em repouso.^6^

De acordo com o mecanismo, quanto maior o volume diastólico ventricular, mais as fibras miocárdicas são distendidas durante a diástole. Dentro de uma faixa fisiológica normal, quanto mais as fibras miocárdicas são distendidas, maior a tensão nas fibras musculars e maior a força de contração ventricular quando estimuladas.^6^

Holubarsch et al.,^7^ descobriram que o mecanismo de Frank-Starling é mantido no estágio final da insuficiência de corações humanos, enquanto alterações significativas da distensibilidade miocárdica diastólica são evidentes na insuficiência cardíaca crônica.^7^

A ecocardiografia tridimensional pode medir com precisão a pré-carga de forma não invasiva, e a curva volume-tempo pode calcular o fluxo em qualquer estágio do ciclo cardíaco.^8,9^

Hammermeister et al.,^10^ validaram de forma invasiva essa medida em 1974. A taxa de ejeção sistólica do VE de pico (S dV/dt) foi calculada a partir de um único plano e os volumes do VE medidos cineangiograficamente em 113 pacientes adultos e relacionados a outras medidas de função cardiovascular. A média de S dV/dt para o grupo de 29 pacientes normais não foi significativamente diferente em pacientes com doença arterial coronariana, estenose aórtica, estenose mitral ou cardiomiopatia. S dV/dt correlacionou-se mal com a fração de ejeção e a pressão diastólica final do VE.^10^

Este estudo mostra que o fluxo sistólico instantâneo e o volume sistólico foram semelhantes entre pacientes com disfunção ventricular grave por CC e controles saudáveis. O grande mérito da metodologia é o emprego pioneiro desta ferramenta não invasiva em CC.

Eles demonstraram e confirmaram que o aumento do volume diastólico final do VE em pacientes com CC é o principal mecanismo de adaptação que mantém o fluxo e os volumes sistólicos na disfunção sistólica grave.

A relação QS/Volume diastólico final do VE representa a função sistólica global do ventrículo esquerdo. Mais estudos são recomendados para confirmar a utilidade e valor prognóstico desses achados na melhoria do manejo clínico de pacientes com CC.
