# Supplementary material for: É Possível Estudar de Forma não Invasiva as Adaptações Hemodinâmicas da Cardiomiopatia Chagásica pela Curva Volume-Tempo Utilizando a Ecocardiografia 3D?
Source: Arq Bras Cardiol. 2022 Jun 6;118(6):1106–7. [Article in Portuguese] doi: 10.36660/abc.20220284 (PMC9345163; doi:10.36660/abc.20220284)
Supplement: Translation

# Translation: Is it Possible to Non-Invasively Study the Hemodynamic Adaptations of Chagas Cardiomyopathy by the Volume-Time Curve Using 3D Echocardiography?

**Authors:** José Luiz Barros Pena

**Affiliations:** 1 Belo Horizonte MG Brazil Faculdade Ciências Médicas de Minas Gerais, Belo Horizonte, MG – Brazil; 2 Belo Horizonte MG Brazil Hospital Felício Rocho, Belo Horizonte, MG – Brazil

**Keywords:** Echocardiography, Three-Dimensional/methods, Heart Failure, Chagas Caddiomyopathy, Stroke Volume, Blood Pressure

Three-dimensional echocardiography (3DE) represents a great innovation in cardiovascular ultrasound.^1^ Increased computer processing power and advances in the development of transducers have allowed acquiring of cardiac structures from any spatial point of view, without assumptions about their shape. Recent studies have demonstrated that when cardiac chamber sizes are quantified using 3DE, their volumes are similar to those obtained using cardiac magnetic resonance imaging compared to two-dimensional echocardiography (2DE).^2,3^ The usefulness of 3DE has particularly been demonstrated primarily in realistic anatomical heart valve images and in guiding and monitoring cardiac procedures.^4^

3DE allows volume calculation of the left ventricle (LV) throughout the cardiac cycle, making it possible to construct a volume-time curve. This method is more accurate than 2DE because the left ventricular volume is constructed by analyzing hundreds of points at the edge of the endocardium. No specific plane or geometric model is necessary to describe the complex LV structure. In this paper, Pinto et al.^5^ tested the hypothesis of studying the hemodynamic adaptations of non-invasive Chagas cardiomyopathy using the volume-time curve generated by 3DE.^5^ They generated a polynomial adjusted to the LV volume curve using specific software. Their objective was to present a cross-sectional study evaluating LV function, comparing volume curves in 20 patients with Chagas cardiomyopathy (CC) and 15 gender- and age-matched healthy controls.

The CC patients presented greater LV end-diastolic and end-systolic volumes and lowered LV ejection fraction than the control group. However, the stroke volume and maximum ejection flow during systole, QS, were similar between groups. A strong correlation existed between flow and stroke volumes, Rs=0.91, p<0.001.

The CC group presented a lower QS/LV end-diastolic volume ratio than the control. The QS/LV end-diastolic volume ratio presented a strong correlation with ejection fraction, Rs=0.89, p<0.001.

The maximum flow in the early and passive filling phases, QE, and during atrial contraction, QA, was similar between patients and controls.

Although the CC patients had severe LV systolic dysfunction with a 30% ejection fraction, the stroke volumes were similar to controls.^5^

Any LV with a low ejection fraction but increased end-diastolic volume ejects the same amount of blood as a LV with normal end-diastolic volume and ejection fraction. This difference occurs due to the preservation of the Frank-Starling mechanism in CC patients at rest.^6^

According to the mechanism, the greater the ventricular diastolic volume, the more the myocardial fibers are stretched during diastole. Within a normal physiologic range, the more the myocardial fibers are stretched, the greater the tension in the muscle fibers and the greater the ventricular contraction force when stimulated.^6^

Holubarsch et al.^7^ found that the Frank-Starling mechanism is maintained in the end-stage of failing human hearts, whereas significant alterations of diastolic myocardial distensibility are evident in chronic heart failure.^7^

Three-dimensional echocardiography can accurately measure a non-invasive preload, and the volume-time curve can calculate flow at any stage of the cardiac cycle.^8,9^

Hammermeister et al.^10^ invasively validated this measure in 1974. Peak LV systolic ejection rate (S dV/dt) was calculated from a single plane, and cineangiographically measured LV volumes in 113 adult patients and related to other measures of cardiovascular function. Mean S dV/dt for the group of 29 normal patients was not significantly different in patients with coronary artery disease, aortic stenosis, mitral stenosis, or cardiomyopathy. S dV/dt correlated poorly with the ejection fraction and LV end-diastolic pressure.^10^

This study shows that instantaneous systolic flow and stroke volume were similar between patients with severe ventricular dysfunction due to CC and healthy controls. The great merit of the methodology is the first usage of a non-invasive tool in CC.

They demonstrated and confirmed that an increase in LV end-diastolic volume in CC patients is the main adaptation mechanism maintaining flow and stroke volumes in severe systolic dysfunction.

This study showed the QS/LV end-diastolic volume to represent LV global systolic function. Further studies are recommended to confirm the usefulness and prognostic value of these findings in improving the clinical management of CC patients.
